# Hsa_circ_0000520 Promotes Non-Small Cell Lung Cancer Progression through the miR-1258/AKT3 Axis

**DOI:** 10.1155/2022/3676685

**Published:** 2022-12-24

**Authors:** Lan Han, Hanxue Yang, Wei Wei, Fen Hu, Limei Yuan

**Affiliations:** Department of Oncology, Xiangyang Central Hospital, Affiliated Hospital of Hubei University of Arts and Sciences, Xiangyang 441021, Hubei, China

## Abstract

**Background:**

There are several previous studies suggesting that circular RNAs (circRNAs) are involved in tumorigenesis of non-small cell lung cancer (NSCLC). Nevertheless, the role of circRNA_0000520 (circ_0000520) in this disease has not yet been studied.

**Methods:**

circ_0000520, microRNA (miR)-1258, and AKT serine/threonine kinase 3 (AKT3) mRNA expression levels were detected by qPCR. CCK-8, EdU, and Transwell assays were utilized to detect NSCLC cells' malignant biological behaviors. The targeted relationship between miR-1258 and AKT3 3′-UTR or circ_0000520 was verified through the dual-luciferase reporter gene assay. Western blotting was utilized to measure the AKT3 expression after circ_0000520 and miR-1258 were selectively regulated.

**Results:**

circ_0000520 was upregulated in NSCLC. Highly expressed circ_0000520 is linked to the NSCLC patient's advanced TNM stage and lymph node metastasis. circ_0000520 overexpression facilitated NSCLC cell growth, migration, and invasion. miR-1258 was identified as the downstream target of circ_0000520. miR-1258 overexpression weakened the effect of circ_0000520 overexpression on NSCLC cells. miR-1258 targeted and inhibited AKT3. circ_0000520 positively regulated the AKT3 expression in NSCLC cells by sponging miR-1258.

**Conclusion:**

circ_0000520 upregulates AKT3 by competitively binding with miR-1258 to facilitate NSCLC progression.

## 1. Introduction

Globally, lung carcinoma is the biggest cause of cancer-related deaths [1]. Non-small cell lung cancer (NSCLC) makes up over 80% of lung cancer cases [2]. At present, the common treatment strategies are surgery, chemotherapy, radiotherapy, targeted therapy, and immunotherapy [3]. Despite recent improvements in its diagnosis and therapy strategies, NSCLC patients' prognosis is still adverse [4, 5].

In the last several decades, more and more non-coding RNAs (ncRNAs) have been discovered and investigated [6, 7]. With a covalently closed-loop structure, circular RNAs (circRNAs) have neither a 5′ end cap nor a 3′ end poly(A) tail [8]. circRNAs are very stable and are transcribed in a tissue-specific manner [9]. Besides, circRNAs participate in tumorigenesis and may act as biomarkers [10]. Reportedly, multiple circRNAs are dysregulated in NSCLC and can promote or inhibit NSCLC progression. For instance, circ_100395 overexpression suppresses the malignancy of NSCLC cells [11]. In NSCLC tissues, circ_POLA2 is highly expressed and high circ_POLA2 expression is associated with NSCLC patients' poor prognosis [12]. circ_0000520 is downregulated in gastric carcinoma and breast carcinoma and can act as a new biomarker [13, 14]. Nevertheless, the functions of circ_0000520 in NSCLC tumorigenesis warrant further elucidation.

Known as highly conserved small ncRNAs, microRNAs (miRNAs) bind to mRNA 3′-UTR to result in mRNA degradation or translation inhibition, thus modulating posttranscriptional gene expression [15]. A lot of miRNAs serve as tumor-suppressive factors or oncogenic factors to partake in tumorigenesis, development, recurrence, and metastasis [16]. There are increasing studies showing that miRNAs participate in NSCLC tumorigenesis and development [17–21]. For instance, miR-451a targets ATF2 to repress the aggressiveness of NSCLC cells [20]. In NSCLC tissues and cells, miR-1258 is downregulated and it suppresses NSCLC progression via the GRB2/Ras/Erk axis [22]. Nonetheless, the mechanism of miR-1258 dysregulation in NSCLC is yet to be clarified.

The present work investigated the expression pattern of circ_0000520 and subsequently explored the exact role of the circ_0000520/miR-1258/AKT serine/threonine kinase 3 (AKT3) regulatory axis in NSCLC. Our work broadens our understanding of NSCLC's pathogenesis and provides potential biomarkers for the disease.

## 2. Materials and Methods

### 2.1. Ethical Statement and Patient Tissues

Thirty-seven pairs of tumorous tissues and para-tumorous tissues of NSCLC patients surgically resected at Xiangyang Central Hospital were collected. None of the patients received neoadjuvant therapy. This study was performed with each patient's informed consent. The procedures of the present work were approved by the Ethics Committee of Xiangyang Central Hospital. NSCLC patients' clinical features are described in Table 1.

### 2.2. Cell Culture

From American Type Culture Collection, NSCLC cell lines (NCI-H1299, A549, H460, NCI-H2106, and H1975) and immortalized bronchial epithelial cells (BEAS-2B) were bought. All of the cells were cultured in DMEM medium (HyClone) with 100 U/ml penicillin and 100 *μ*g/ml streptomycin (Invitrogen) and 10% fetal bovine serum (FBS; Sigma), which was then placed in an incubator in 5% CO_2_ at 37°C. When the cells grew to 70–80% confluency, 0.25% trypsin (Roche) was used for subculture. GenePharma was the provider of circ_0000520 overexpression plasmid (pcDNA3.1-circ _0000520), negative control plasmid (pcDNA3.1-NC), miR-1258 mimics, and the control (miR-NC). Lipofectamine® 3000 (Invitrogen) was used for transfecting the abovementioned plasmids and oligonucleotides into A549 and H460 cells. 24 h later, the efficiency of cell transfection was determined through quantitative real-time polymerase chain reaction (qPCR).

### 2.3. qPCR

TRIzol reagent (Invitrogen) was adopted to isolate the total RNA. A PrimeScript RT kit (TaKaRa) was used for the reverse transcription. On an ABI 7900 fast real‐time PCR system (Applied Biosystems), qRT‐PCR was conducted utilizing a SYBR Green Master Mix II kit (TaKaRa). The expression of GAPDH was adopted to normalize the expression levels of mRNA and circ_0000520, and the expression of miRNA was normalized with small RNA RNU6B (U6). The relative expression of genes was quantified through the 2^−ΔΔCT^ method. Check Table 2 for the sequences of the primers.

### 2.4. Nucleocytoplasmic Separation Experiment

A PARIS™ kit (ThermoFisher) was applied for carrying out the nucleocytoplasmic separation experiment. The TRIzol method was used to extract the cytoplasmic and nuclear RNA, and then, qPCR was conducted to examine the circ_0000520 expression in the nucleus and cytoplasm, respectively. U6 and GAPDH functioned as the controls of subcellular localization.

### 2.5. Cell Counting Kit-8 (CCK-8) Assay

The NSCLC cells were inoculated into 96-well plates (2 × 10^3^ cells/well). Then, 10 *μ*L of CCK-8 solution (MedChemExpress) was supplemented into each well at different time points, and the cells were incubated at 37°C for another 2 h. After terminating the culture, the absorbance values were measured at a wavelength of 450 nm.

### 2.6. 5‐Ethynyl‐2′‐Deoxyuridine (EdU) Assay

An EdU detection kit (RiboBio) was used to detect cell proliferation. H460 and A549 cells were cultured for 24 h. The cells were then treated with 50 *μ*M EdU at 37°C for 2 h. Next, the culture solution was discarded, and subsequently, the A549 and H460 cells were fixed for 30 min with 4% paraformaldehyde. Next, 0.5% Triton X-100 was applied to increase the permeability, and then, the A549 and H460 cells were incubated with Apollo fluorescence staining reaction solution for 30 min in a dark place. Then, the cells were stained for 15 min with DAPI staining solution. Finally, the cells were placed under a fluorescence microscope (magnification of 200) (Olympus), and the EdU-positive cells were counted.

### 2.7. Transwell Assays

A549 and H460 cells, after being digested with 0.25% trypsin, were centrifuged and resuspended in a serum-free medium. Matrigel (pore size: 8 *µ*m; 1 : 10; BD Biosciences) was only used for invasion assay. A549 and H460 cells (5 × 10^4^) were added to the top compartment of Transwell, and 10% FBS-containing DMEM was added to the bottom chamber, and the cells were cultured for 24 h at 37°C. Subsequently, the cells that had failed to migrate were discarded; the cells that had migrated were fixed for 10 min with 4% paraformaldehyde and subsequently stained with 0.5% crystal violet solution. Under an inverted microscope (Olympus), the cells were counted.

### 2.8. Dual-Luciferase Reporter Gene Assay

From Promega (Madison), all luciferase reporter vectors circ_0000520 mutant (MUT), circ_0000520 wild type (WT), AKT3 MUT, and AKT3 WT were obtained. Next, circ_0000520 WT/MUT or AKT3 WT/MUT and miR-1258 mimics or its negative control were co-transfected into H460 and A549 cells. The luciferase activity was measured at 48 h after the transfection.

### 2.9. Immunoblotting

The transfected cells were lysed by RIPA buffer (Beyotime). After centrifugation, the cell supernatant was collected. The supernatant was then heated in a 100°C water bath for 10 min for denaturing the protein. Then, the protein was isolated by SDS-PAGE and transferred to the polyvinylidene fluoride (PVDF) membrane (Millipore). The membrane was blocked at room temperature with 5% skimmed milk for 1 h and subsequently rinsed with Tris-buffered saline with Tween-20 (TBST) 3 times. Subsequently, the membrane and primary antibodies (anti-AKT3 antibody (ab152157, 1 : 1000, Abcam) and anti-GAPDH antibody (ab245355, 1 : 1000, Abcam)) were incubated at 4°C overnight. After TBST rinsing, the membrane and goat anti-rabbit IgG H&L (ab205718, 1 : 5000, Abcam) were incubated for 1 h at room temperature. GAPDH acted as the internal control. The ECL chemiluminescence kit (Promega) was utilized for developing the bands.

### 2.10. Transplanted Tumor Experiment

This experiment was supported by the Animal Research Ethics Review Board of Xiangyang Central Hospital. Sixteen nude mice (4 weeks old, bought from Model Animal Center of Wuhan University, Wuhan, China) were randomly divided into two groups (8 mice per group). Subsequently, the transfected A549 cells were respectively inoculated into the back of the nude mice. Three weeks later, mice were sacrificed with euthanasia and the volume of the tumor in the two groups was compared.

### 2.11. Statistical Analysis Technique

Each assay was conducted in triplicates and repeated 3 times. SPSS21.0 (SPSS Inc.) was adopted to statistically analyze the data. “*x* ± *s*” was used to represent the data. *t*-test and one-way analysis of variance were performed to compare the means of 2 and more groups, respectively. Fisher's exact test was utilized to analyze the correlation of circ_0000520 expression with NSCLC patients' clinical parameters. Pearson correlation analysis was conducted to assess the correlation. A difference was of statistical significance when *P* < 0.05.

## 3. Results

### 3.1. In NSCLC Tissues and Cells, circ_0000520 Is Upregulated

**Figure 1:**
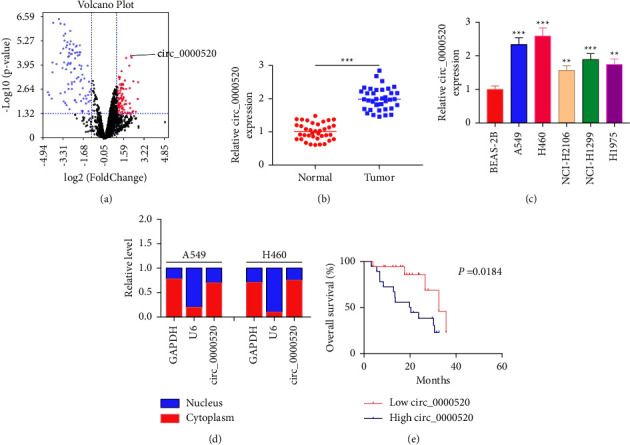
Circ_0000520 is upregulated in NSCLC tissues and cells. (a) The volcano plot displays the expression differences of circRNAs in the data set GSE158695 (cutoff criterion: log_2_|fold change| > 1 and *P* < 0.05). The downregulated circRNAs are marked in blue, and the upregulated circRNAs are marked in red. Black represents circRNAs with no significant difference. (b) Detection of circ_0000520 expression in NSCLC tissues and para-cancerous tissues by qPCR. (c) Detection via qPCR of circ_0000520 expression in NSCLC cell lines (A549, H460, NCI-H2106, NCI-H1299, and H1975) and human immortalized bronchial epithelial cells (BEAS-2B). (d) Nucleocytoplasmic separation experiment was performed to detect the subcellular localization of circ_0000520 in NSCLC cells. AuthorAnonymous. (e) Kaplan–Meier survival analysis was conducted to examine the correlation between the circ_0000520 expression and the overall survival rate of NSCLC patients. ^*∗∗*^*P* < 0.01 and ^*∗∗∗*^*P* < 0.001.

Through the analysis of the microarray data, the dataset GSE158695, it was revealed that circ_0000520 is upregulated in NSCLC tissues (Figure 1(a)). It was also revealed that as opposed to para-cancerous tissues or human immortalized bronchial epithelial cells (BEAS-2B), circ_0000520 was significantly upregulated in NSCLC (Figures 1(b) and 1(c)). Furthermore, circ_0000520 was primarily located in the cytoplasm (Figure 1(d)). To assess the correlation of 37 NSCLC patients' clinicopathological features with circ_0000520 expression, the patients were divided into high (*n* = 18) and low (*n* = 19) expression groups. It was revealed that high circ_0000520 expression was strongly associated with the NSCLC patients' lymph node metastasis and a higher TNM stage (Table 1). Additionally, a high circ_0000520 expression was positively correlated with low overall survival rate of the patients (Figure 1(e)).

### 3.2. Impacts of the Overexpression of circ_0000520 on NSCLC Cells

**Figure 2:**
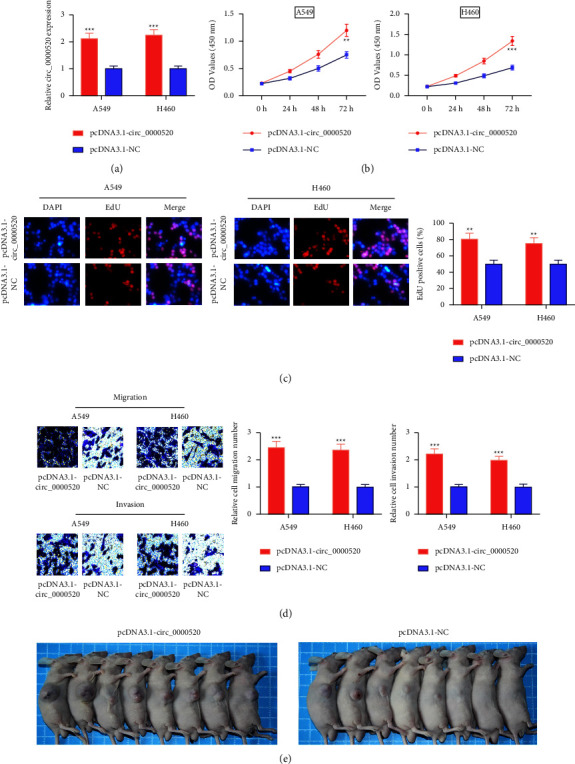
Circ_0000520 promotes NSCLC cell proliferation, migration, and invasion. (a) After transfecting pcDNA3.1-circ_0000520 or pcDNA3.1-NC into A549 and H460 cells, qPCR was carried out to detect the circ_0000520 expression in A549 and H460 cells. (b, c) CCK-8 method and EdU assay were utilized to detect the effect of circ_0000520 overexpression on A549 and H460 cell proliferation. (d) Transwell assays were utilized to detect the effects of circ_0000520 overexpression on A549 and H460 cell migration and invasion. (e) A transplanted tumor model was used to evaluate the effect of circ_0000520 overexpression on tumor growth *in vivo*. ^*∗∗*^*P* < 0.01 and ^*∗∗∗*^*P* < 0.001.

To study circ_0000520s role in NSCLC, circ_0000520 overexpression plasmids were transfected into A549 and H460 cells (Figure 2(a)). circ_0000520 overexpression markedly enhanced A549 and H460 cell growth (Figures 2(b) and 2(c)). Additionally, circ_0000520 overexpression can remarkably promote A549 and H460 cell migration and invasion (Figure 2(d)). Additionally, *in vivo* experiments suggested that high expression of circ_0000520 promoted the growth of tumor cells which were transplanted into the nude mice (Figure 2(e)). The aforementioned findings indicate that circ_0000520 might be an oncogene in NSCLC progression.

### 3.3. circ_0000520 Adsorbs miR-1258

**Figure 3:**
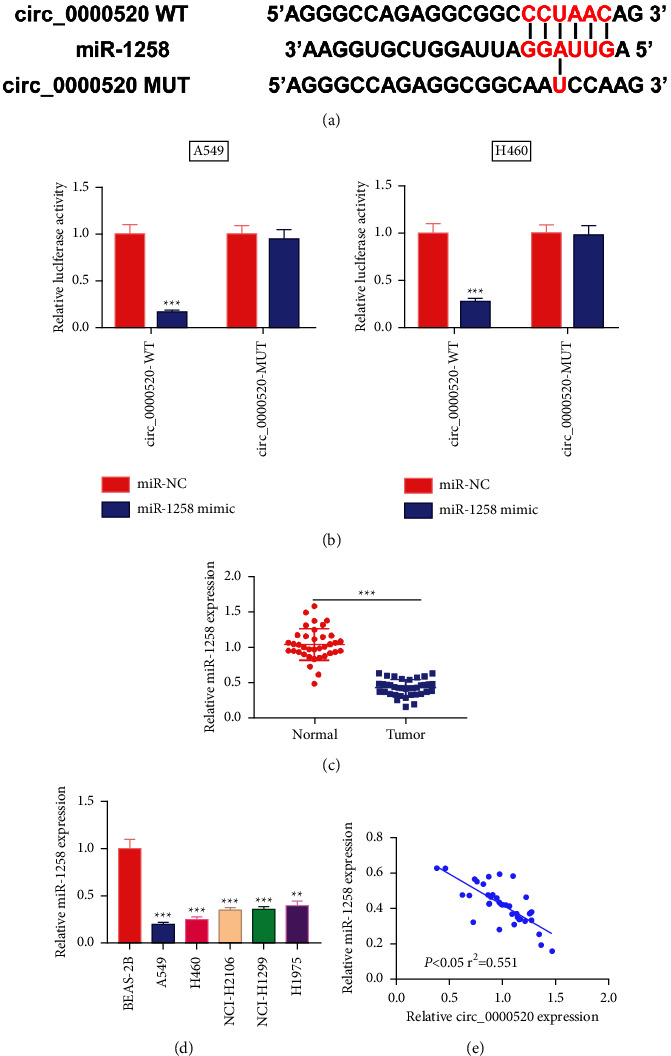
Circ_0000520 adsorbs miR-1258. (a) The CircInteractome database was employed to predict the binding site between circ_0000520 and miR-1258. AuthorAnonymous. (b) Dual-luciferase reporter gene assay was conducted to verify the binding relationship between circ_0000520 and miR-1258. (c, d). qPCR was performed to detect the miR-1258 expression in NSCLC tissues and cells. (e) Pearson's correlation analysis of the correlation between circ_0000520 and miR-1258 expressions in NSCLC tissues. ^*∗∗*^*P* < 0.01 and ^*∗∗∗*^*P* < 0.001.

Next, we searched CircInteractome online website to predict circ_0000520s potential target miRNAs, and observed that there was a binding sequence between circ_0000520 and miR-1258 (Figure 3(a)). Overexpression of miR-1258 markedly repressed circ_0000520 WT's luciferase activity in H460 and A549 cells, with no obvious influence on that of circ_0000520 MUT (Figure 3(b)). Next, qPCR indicated that unlike para-cancerous tissues or BEAS-2B cells, miR-1258 expression was underexpressed in the cancer group (Figures 3(c) and 3(d)). Notably, circ_0000520 expression and miR-1258 expression were in negative correlation in the tissue samples (Figure 3(e)).

### 3.4. Circ_0000520 Plays a Role by Targeting miR-1258

**Figure 4:**
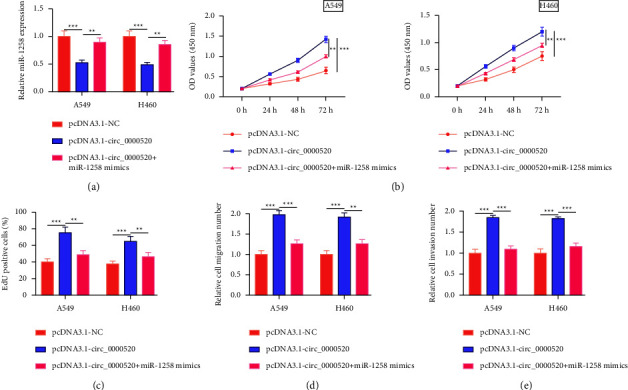
Circ_0000520 promotes NSCLC progression by targeting miR-1258. (a) PcDNA3.1-NC, pcDNA3.1-circ_0000520, and pcDNA3.1-circ_0000520+miR-1258 mimics were transfected into A549 and H460 cells, separately, and miR-1258 expression in A549 and H460 cells after transfection was detected by qPCR. (b, c) A549 and H460 cell proliferation after transfection were detected by CCK-8 method and EdU assay. (d, e) Detection of A549 and H460 cell migration and invasion abilities after transfection through Transwell assays. ^*∗∗*^*P* < 0.01 and ^*∗∗∗*^*P* < 0.001.

Next, “rescue” experiments were performed. It was revealed that circ_0000520 overexpression would suppress miR-1258 expression in H460 and A549 cells, whereas transfection of miR-1258 mimics would reverse this effect (Figure 4(a)). Accordingly, exogenous expression of miR-1258 mimics markedly decreased the promoting effect that overexpression of circ_0000520 on A549 and H460 cells (Figures 4(b)–4(e)).

### 3.5. circ_0000520 Regulates the AKT3 Expression in NSCLC Cells by Repressing miR-1258

**Figure 5:**
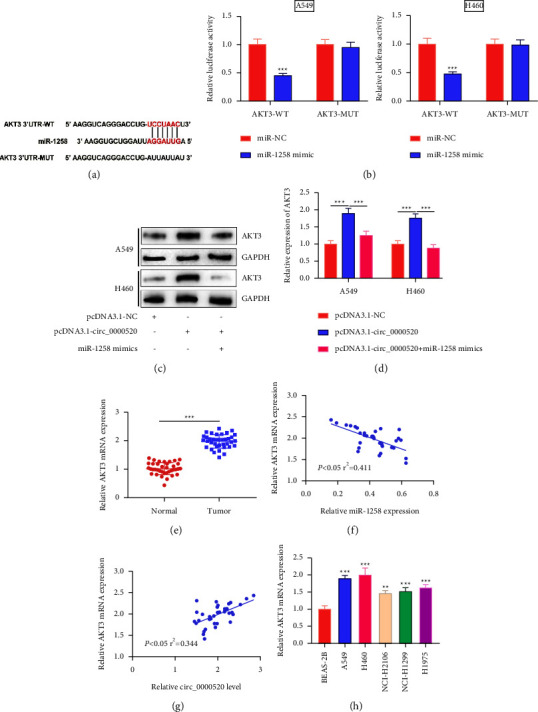
Circ_0000520 plays a part by regulating the miR-1258/AKT3 axis. (a) The binding site between miR-1258 and AKT3 3′-UTR was predicted by the TargetScan database. (b) Dual-luciferase reporter gene assay was utilized to verify the binding relationship between miR-1258 and AKT3 3′-UTR. (c, d) Western blot was conducted to detect the level of AKT3 protein in A549 and H460 cells transfected with pcDNA3.1-NC, pcDNA3.1-circ_0000520, and pcDNA3.1-circ_0000520+miR-1258 mimics. (e) Detection by qPCR of the AKT3 mRNA expression in NSCLC tissues and para-cancerous tissues. (f, g) Pearson's correlation analysis of the correlation between the AKT3 mRNA expression and miR-1258 or circ_0000520 expression in NSCLC tissues. (h) qPCR was utilized to detect AKT3 mRNA expression in NSCLC cell lines and BEAS-2B cells. ^*∗∗*^*P* < 0.01 and ^*∗∗∗*^*P* < 0.001.

miR-1258's downstream target genes were predicted utilizing the TargetScan database, and it was revealed that AKT3 has a binding site to miR-1258 (Figure 5(a)). Overexpression of miR-1258 inhibited AKT3 WT's luciferase activity (Figure 5(b)). Next, we found that upregulating circ_0000520 promoted the AKT3 protein expression, while upregulating miR-1258 could reduce this effect (Figures 5(c) and 5(d)). Furthermore, the AKT3 mRNA expression was markedly elevated in NSCLC (Figure 5(e)). The expression levels of miR-1258 and AKT3 mRNA were in negative correlation (Figure 5(f)) and those of AKT3 mRNA and circ_0000520 expression were in positive correlation (Figure 5(g)). Unlike BEAS-2B cells, AKT3 mRNA expression was enhanced in NSCLC cell lines (Figure 5(h)).

## 4. Discussion

A growing amount of evidence shows that the expression characteristics of circRNAs are closely associated with the adverse clinical parameters of patients, and circRNA dysregulation often promotes different malignant behaviors [23, 24]. For instance, in NSCLC, circ_100395 is underexpressed, and circ_100395 overexpression represses the malignancy of NSCLC cells [25]. circ-ACACA is upregulated in NSCLC and knocking down circ-ACACA suppresses NSCLC development by sponging miR-1183 and regulating the PI3K/PKB axis [26]. This study reports that circ_000052 is dysregulated in NSCLC by analyzing the circRNA microarray. Furthermore, it is unveiled that high circ_0000520 expression is correlated with adverse prognosis of NSCLC patients. Moreover, we observe that circ_0000520 increases the malignancy of NSCLC cells. In a nutshell, the aforementioned evidence demonstrates that circ_0000520 may act as an oncogene in NSCLC.

The role of miR-1258 in tumorigenesis has been widely reported in recent years [27, 28]. In breast carcinoma cells, miR-1258 expression is reduced and miR-1258 can downregulate E2F1 expression [29]. In cervical cancer cells, miR-1258 is downregulated and it inhibits the malignancy biological behaviors of cancer cells by modulating E2F1/P53 signaling [30]. circRNAs can function as miRNAs sponges in many cancers [31, 32]. For instance, circ_0000326 accelerates lung adenocarcinoma progression via sponging miR-338-3p [33]. circ_0046264 suppresses the malignancy of lung carcinoma cell by acting on the miR-1245/BRCA2 axis [34]. This study revealed that circ_0000520 can sponge miR-1258 in NSCLC cells. Additionally, miR-1258 upregulation reversed the promotional impact that circ_0000520 overexpression had on the malignancy of NSCLC cells, suggesting circ_0000520 may regulate NSCLC development via sponging miR-1258. Previous studies have reported that some miRNAs are target miRNAs of circ_0000520, such as miR-512-5p, miR-1296, miR-556-5p, and so on [35–37]. Our study identified a novel downstream miRNA target of circ_0000520 and miR-1258.

Known as a serine/threonine protein kinase, AKT3 is pivotal in modulating cell proliferation, differentiation, apoptosis, and migration [38]. AKT3 is abnormally expressed in various cancers and affects cancer progression. For example, in papillary thyroid carcinoma tissues and cells, AKT3 expression is elevated; miR-203 represses the malignancy of papillary thyroid cancer cells by downregulating AKT3 [39]. There is a study showing that AKT3 can promote prostate cancer cell proliferation by regulating Akt, B-Raf, and TSC1/TSC2 [40]. Besides, miR-217 can suppress NSCLC development by reducing the AKT3 expression [41]. This study revealed that AKT3 is miR-1258's downstream target in NSCLC cells. Moreover, AKT3 expression is modulated by the circ_0000520/miR-1258 axis. The abovementioned evidence demonstrates that circ_0000520 regulates NSCLC development through regulating the miR-1258/AKT3 axis.

To sum up, circ_0000520 expression in NSCLC is elevated, and it enhances the malignancy of cancer cells. In terms of mechanism, circ_0000520 increases AKT3 expression via absorbing miR-1258. These findings may provide innovative ideas for NSCLC treatment.

## Figures and Tables

**Table 1:** Correlation between the circ_0000520 expression and clinicopathologic parameters of 37 NSCLC patients.

Clinicopathologic parameter	Number (*n* = 37)	*circ_0000520*		*P* value
		High (*n* = 18)	Low (*n* = 19)	
*Age (years)*				1.000
≥60	17	8	9	
<60	20	10	10	
*Gender*				0.380
Male	27	15	12	
Female	20	8	12	
*Tumor size (cm)*				0.080
≥2 cm	26	16	10	
<2 cm	21	7	14	
*TNM stage*				0.029^*∗*^
I/II	11	2	9	
III/IV	26	16	10	
*Smoking status*				0.456
Non-smoker	10	5	5	
Smoker	13	8	5	
Heavy smoker	14	5	9	
*Lymph node metastasis*				0.038^*∗*^
Absent	13	3	10	
Present	24	15	9	

^*∗*^ *P* < 0.05 (by Fisher exact test).

**Table 2:** The list of primers used in this study.

Primer	Sequence (5′-3′)
circ_0000520	Forward: GTCTGAGACTAGGGCCAGAGGC
	Reverse: GACATGGGAGTGGAGTGACAGG

miR-1258	Forward: AGTTAGGATTAGGTCGTGGAA
	Reverse: GCGAGCA CAGAATTAATACGAC

GAPDH	Forward: TCACCAGGGCTGCTTTTAAC
	Reverse: TGACGGTGCCATGGAATTTG

U6	Forward: GGAATGCTTCAAAGAGTTGTG
	Reverse: ATACAGAGAAAGTTAGCACGG

AKT3	Forward: TGTGGATTTACCTTATCCCCTCA
	Reverse: GTTTGGCTTTGGTCGTTCTGT

## Data Availability

The data used to support the findings of this study are available from the corresponding author upon request.
